# The Evaluation of Meloxicam Nanocrystals by Oral Administration with Different Particle Sizes

**DOI:** 10.3390/molecules27020421

**Published:** 2022-01-10

**Authors:** Yao Yu, Yang Tian, Hui Zhang, Qingxian Jia, Xuejun Chen, Dongzhou Kang, Yimeng Du, Shenghan Song, Aiping Zheng

**Affiliations:** 1 Pharmaceutical Experiment Center, College of Pharmacy, Yanbian University, Yanji 133002, China; yuyaottt@163.com (Y.Y.); jiaqingxian2021@163.com (Q.J.); YukiCcCx@163.com (X.C.); 2 State Key Laboratory of Toxicology and Medical Countermeasures, Beijing Institute of Pharmacology and Toxicology, 27th Taiping Road, Haidian District, Beijing 100850, China; tianyang1127@126.com (Y.T.); zhhui58@126.com (H.Z.); 3 Department of Vascular Surgery, Beijing Chaoyang Hospital, Capital Medical University, Beijing 100020, China

**Keywords:** meloxicam, nanocrystal, particle sizes, molecular simulation, pharmacokinetic

## Abstract

Meloxicam (MLX) is a non-steroidal anti-inflammatory drug used to treat rheumatoid arthritis and osteoarthritis. However, its poor water solubility limits the dissolution process and influences absorption. In order to solve this problem and improve its bioavailability, we prepared it in nanocrystals with three different particle sizes to improve solubility and compare the differences between various particle sizes. The nanocrystal particle sizes were studied through dynamic light scattering (DLS) and laser scattering (LS). Transmission electron microscopy (TEM) was used to characterize the morphology of nanocrystals. The sizes of meloxicam-nanocrystals-A (MLX-NCs-A), meloxicam-nanocrystals-B (MLX-NCs-B), and meloxicam-nanocrystals-C (MLX-NCs-C) were 3.262 ± 0.016 μm, 460.2 ± 9.5 nm, and 204.9 ± 2.8 nm, respectively. Molecular simulation was used to explore the distribution and interaction energy of MLX molecules and stabilizer molecules in water. The results of differential scanning calorimetry (DSC) and powder X-ray diffraction (PXRD) proved that the crystalline state did not change in the preparation process. Transport studies of the Caco-2 cell model indicated that the cumulative degree of transport would increase as the particle size decreased. Additionally, plasma concentration–time curves showed that the AUC_0–∞_ of MLX-NCs-C were 3.58- and 2.92-fold greater than those of MLX-NCs-A and MLX-NCs-B, respectively. These results indicate that preparing MLX in nanocrystals can effectively improve the bioavailability, and the particle size of nanocrystals is an important factor in transmission and absorption.

## 1. Introduction 

Increasing the solubility and bioavailability of poorly soluble drugs remains a challenging task. The absorption of oral drugs is a key physiological process. Drugs can only be absorbed effectively when they are dissolved in the body. Therefore, solubility limits the absorption process in poorly soluble drugs. Meloxicam (MLX) is a non-steroidal anti-inflammatory drug (NASID) which was developed by Boehringer Ingleheim for the treatment of rheumatoid arthritis and osteoarthritis [1]. MLX is classified as a Class II drug according to the Biopharmaceutical Classification System (BCS) due to its low solubility and high permeability [2]. MLX is a weakly acidic drug, with its solubility depending on pH and relating to its multiple ionization states [3]. However, the low dissolution and solubility of MLX (about 4.4 μg/mL in water) [4] greatly limits its bioavailability. Therefore, due to its low solubility and slow oral absorption, the pharmaceutical effects of MLX are slow. In order to improve the bioavailability of MLX, researchers have developed many strategies to improve drug solubility, including salt formulation, amorphous solid dispersion techniques [5,6], forming MLX into self-emulsifying drug delivery systems, etc. However, these methods have only been used for a certain number of drugs, and other compounds may be introduced during the preparation process. More importantly, there is the limitation of drug loading. 

**Figure 1:**
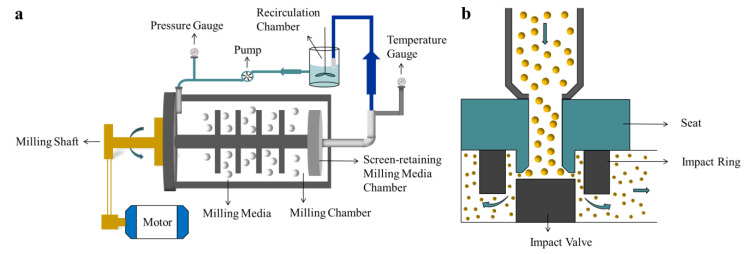
Schematic illustration of the top-down process: (**a**) wet-milling technology; (**b**) high-pressure homogenization technology.

Nanocrystal technology has attracted widespread attention in increasing the dissolution rate of poorly soluble drugs. Currently, there are a variety of nanocrystal products available on the market, and the technology has been widely used in the development of NSAIDs [7,8,9,10,11,12]. Nanocrystals were originally intended to improve the bioavailability of poorly water-soluble drugs [13]; these are defined as drug crystals smaller than several micrometers in diameter [14]. As the specific surface area of the drug is increased, the solubility and dissolution rate of the drug can clearly be improved by nanocrystal drugs. The methods for preparing nanocrystals are mainly divided into top-down and bottom-up techniques [15]. Top-down processes apply external force to the drug to cause the drug particles to become smaller, and include wet-milling technology [16] and high-pressure homogenization technology [17,18], the principles of these two methods are shown in Figure 1. The advantages of top-down techniques are their suitability for industrial production, which can be used to prepare drug nanosuspensions in bulk quantities [19]. In bottom-up techniques, the drug is dissolved in an organic solvent, and the drug solution is added to an anti-solvent containing stabilizer under conditions of high-speed mixing, rapid temperature drop and ultrasound [15]. Ochi et al. [20] prepared micrometer-sized MLX crystals using wet milling with three different stabilizers, and investigated the dissolution behavior of the three crystals; Bolourchian et al. [21] used a cooling and anti-solvent precipitation technique to tailor the dissolution and physicochemical properties of MLX nanocrystals, but did not perform pharmacokinetic tests. However, few studies have fully investigated the absorption of MLX nanocrystals with different particle sizes in the range of nanometers to micrometers in vivo and in vitro. The particle size is an important parameter for nanocrystals, undoubtedly playing an important role in absorption. When the drug particle size is at the nanometer level, the solubility and dissolution rate are significantly improved, and the bioavailability also increases accordingly [22]. The saturation solubility, dissolution rate, and mucosal adhesion of nanocrystals could be affected by particle size. We hypothesize that the smaller the particle size, the greater the degree of absorption. Thus far, many researchers have demonstrated that as the size of nanoparticles decreases, the bioavailability increases accordingly [23,24,25,26]. Therefore, we aim to explore the effect of particle size on the absorption of MLX nanocrystals. In order to develop oral MLX nano-formulations better and explore the effect of particle size in vivo and in vitro, we prepared three nanocrystals with different particle sizes, and used these nanocrystals to carry out transport studies and pharmacokinetic research. At the same time, we used molecular simulations to explore how PVP-k17 and MLX combined in water; therefore, the interaction and energy relationships between MLX and PVP- K17 can be better explained at molecular level.

In this study, MLX nanocrystals with three different particle sizes, measuring from micrometers to nanometers in diameter, were prepared to evaluate the importance of particle size in vivo and in vitro. The MLX nanocrystals were prepared using wet milling technology and high-pressure homogenization technology, respectively. Particle size and the polydispersity index of nanometer crystals (PDI) were tested by dynamic light scattering (DLS), whereas the particle size and span value were measured through a laser scattering (LS) method for micrometer crystals. The morphology of nanocrystals was characterized by transmission electron microscopy (TEM), and the crystallinity was measured by differential scanning calorimetry (DSC) and powder X-ray diffractometry (PXRD). Molecular simulations were performed to confirm the molecule structures and interactions between MLX and PVP-k17 in the water system. The Caco-2 cell model was used in a transport study to simulate the process of nanocrystals in the intestine and determine its permeability in the intestinal tract. Pharmacokinetic profiling of MLX after oral administration of the nanosuspension to rats was evaluated to explore the influence of particle size on pharmacokinetics.

## 2. Materials and Methods

### 2.1. Reagents

MLX (with a purity of up to 99.8%) was purchased from JiangSu Feima Pharmaceutical Co. Ltd. (Jiangsu, China). Polyvinylpyrrolidone-k17 (PVP-k17) was gifted by Beijing Fengli Jingqiu Pharmaceutical Co. Ltd. (Beijing, China). Piroxicam was obtained from Beijing Xian Dai Dong Fang Technological Development Co. Ltd. (Beijing. China). Fetal bovine serum (FBS), streptomycin, penicillin, Hank’s balanced salt solution (HBSS), and phosphate-buffered saline (PBS) were obtained from Beijing Rongxia Biotechnology Co. Ltd. (Beijing, China). HPLC-grade triethylamine and acetonitrile were obtained from Thermo Fisher Scientific (Waltham, MA, USA). Isopropanol and ethyl acetate were purchased from Sinopharm Chemical Reagent Co. Ltd. (Shanghai, China).

### 2.2. Animals

Animals were purchased from Beijing Vital River Laboratory Animal Technology Co. Ltd. (Beijing, China). All of the experiments were performed on male SD rats (240–260 g). The rats were allowed one week to adjust to the breeding facility prior to the experiments. Rats were kept in plastic cages under a fixed 12-h light/dark cycle; food and water were freely available. The temperature and humidity in the environment were controlled. Rats were fasted for 12 h with free access to water prior to experiments. All animal experiments complied with ARRIVE guidelines [27] and were approved by the Animal Ethics Committee and the Institutional Animal Care and Use Committee of Beijing Institute of Pharmacology and Toxicology, Beijing, China (IACUC of AMMS-06-2017-001). 

### 2.3. Preparation of Meloxicam Nanoparticles

The preparation methods of three different particle sizes of MLX nanocrystals involved wet-milling technology and high-pressure homogenization technology. Firstly, MLX powder (3%, *w*/*v*) was dispersed in PVPk-17 aqueous solution (1%, *w*/*v*), and the mixture was stirred for 10 min at 1000 rpm by a magnetic stirrer (IKA RO15, Staufen, Germany). It was then stirred for 5 min with an emulsification homogenizer (Fluko FM200A ShangHai, China) at a speed of 3000 rpm to produce a coarse drug suspension. To obtain MLX nanocrystals-A (MLX-NCs-A), a 100 mL drug suspension was placed into the high-pressure homogenizer (ATS Engineering Inc., Brampton, ON, Canada) at 100 bar for 120 circulations. The MLX nanocrystals-B (MLX-NCs-B) and MLX nanocrystals-C (MLX-NCs-C) were prepared with a wet-grinding machine (WAB AK71M-2WKF, Switzerland). The drug suspension circulated for 10 min under conditions of 1500 rpm in order to obtain MLX-NCs-B. For MLX-NCs-C, the drug suspension was firstly circulated for 50 min under conditions of 1500 rpm; next, the rotation speed was changed to 2000 rpm and circulated for 70 min. The cycle time was 120 min in total. Due to the special solubility characteristics of MLX, we used the anionic form in our research [3]. 

### 2.4. High-Performance Liquid Chromatography (HPLC) Analysis

The concentration of MLX was determined with an Agilent 1260 high-performance liquid chromatography system (Agilent Technologies, Boulder, CO, USA) equipped with a diode array detector. Chromatography separation was conducted using a SHISEIDO C18-MGII column (particle size: 5 μm, column dimensions: 4.6 mm ×250 mm). The column temperature was maintained at 25 °C, and the samples were separated using a mobile phase consisting of 0.1 mol/L ammonium acetate and acetonitrile (45/55, *v*/*v*) at a flow rate of 1.0 mL/min, and an automatic injector with an injection volume of 20 μL. The diode array detector was set at a wavelength of 360 nm. The retention time was approximately 7.5 min. 

### 2.5. In Vitro Characterization

#### 2.5.1. Particle Size

Dynamic light scattering (DLS) was used to determine the nanometer particle size and polydispersity index (PDI) of the MLX nanocrystals. A laser scattering (LS) method was used for micrometer crystals. Before the measurement, the MLX nanocrystals were diluted with distilled water to the appropriate scattering intensity. MLX-NCs-A was measured with a Mastersizer 2000 (Malvern Instruments, Royston, UK). Meanwhile, the MLX-NCs-B and MLX-NCs-C were measured by Malvern Zetasizer Nano-ZS90. The results were analyzed by the software provided by Malvern Instruments.

#### 2.5.2. Morphology

The morphology of MLX nanoparticles was studied using transmission electron microscopy (TEM). The nanocrystals were diluted with distilled water and dripped onto the copper grid to dry naturally; the morphology was then observed.

### 2.6. Crystalline Form

#### 2.6.1. Differential Scanning Calorimetry

Differential scanning calorimetry (DSC) was performed in a DSC 200F3 (Netzsch, Germany) instrument to assess the thermal behavior of the samples: raw MLX material, PVP-k17, physical mixture, and three nanocrystals. Samples were sealed in an aluminum pan and heated at 5 °C/min from 50 to 300 °C under a nitrogen atmosphere.

#### 2.6.2. Powder X-ray Diffractometry

Powder X-ray diffractometry (PXRD) analysis was performed using a Bruker D8 Advance with a DaVinci design (Bruker AXS, Madison, WI, USA) to measure the crystalline state of the raw MLX material, PVP-k17, physical mixture, and MLX-NCs-A, MLX-NCs-B and MLX-NCs-C. A 2θ scan range of 5° to 50° at a rate of 10°/min, and a step size of 0.01° per second was utilized. 

### 2.7. Molecular Simulations

In order to explore the intermolecular interaction between MLX and PVP during the preparation of nanocrystals, molecular dynamics simulations were carried out. The simulation results were analyzed with GROMACS 2018 software. A generation amber force field (GAFF) was used for MLX and PVP-k17, with a restrained electrostatic potential (RESP) charge applied to these molecules. In the simulation system, there were 20 molecules in both MLX and PVP samples. The material was solvated in TIP3P water molecules model in a cubic box with sides of 1 nm. First, we performed energy minimization for 5000 steps, and then conducted a short simulation of 100 ps in the NVT and NPT, respectively; finally, the equilibrium simulation of 10 ns was generated. The truncation radius was set as 1.0 nm, and the time step was set as 2 fs. The simulation temperature and pressure were set to 300 K and 1 atmosphere (1.01 bar).

### 2.8. Cytotoxicity of Caco-2 Cell

The cytotoxicity of the 3 different sizes of MLX nanocrystals was measured using an MTT (3-(4,5-dimethylthiazol-2-yl)-2,5-diphenyltetrazolium bromide) assay. The Caco-2 cell line was purchased from Shanghai Fuheng Biotechnology Co. Ltd. (Shanghai, China). Caco-2 cells were seeded in 96-well plates at a density of 5 × 10^4^ cells/well for 24 h, and the plate was incubated in 5% CO_2_ at 37 °C for 24 h. Then, 200 μL MLX nanocrystal (at concentrations of 12.5, 25, 50, 100, 200, and 400 μg/mL) diluent was added to each well and incubated for 3, 6, 12, and 24 h. After the treatment, the wells were washed three times with PBS, and 100 μL 0.5 mg/mL MTT was added to each well and incubated for another 4 h. Then, the supernatant was removed, and 100 μL DMSO was added into each well to dissolve formazan crystals. The absorbance was measured with a multiplate reader (Thermo Scientific, Varioskan LUX, USA) at 490 nm. The cell viability rate was calculated by comparing the absorbance of untreated cells. A medium without MLX nanocrystals was used as a control. 

### 2.9. Transepithelial Penetration of Caco-2 Cells

Caco-2 cells were seeded in 24-well Transwell inserts (0.4 μm pore size, 6.5 mm diameter, Millipore Corporation, Billerica, MA, USA) in RPMI 1640 medium containing 10% FBS, 1× penicillin (100 U/mL) and streptomycin (100 μg/mL) at a seeding density of 5 × 10^4^ cells/well, and incubated for 21 days to obtain an integrated cell monolayer. Transepithelial electrical resistance (TEER) was measured using the Millicell-ERS system (Millipore, Manassas, VA, USA). A TEER of 300 Ω/cm^2^ was selected, indicating the development of functional polarity and an intact monolayer, which is referred to the integrity of the intestinal barrier. First, the medium in the apical side and the basolateral side was discarded, and fresh 37 °C HBSS solution was added for three cycles of washing and equilibration. Then, 0.2 mL of sample was added to the apical side, and 1.0 mL of HBSS was added to the basolateral side. The culture was incubated at 37 °C for 3 h, with 200 μL of sample collected from the basolateral side every 0.5 h and replaced with the same volume of HBSS. Finally, samples in the basolateral side were extracted with acetonitrile and then determined by liquid chromatography–mass spectrometry (LC/MS). The apparent permeability coefficient (*P_app_*) was calculated as follows:

$$P_{app}=\left(\Delta Q/\Delta t\right)/\left(A\times C_{0}\right)$$

where ΔQ/Δt is the amount of MLX transported from the apical side to the basolateral side (in μg); A represents the monolayer area (cm^2^); and $C_{0}$ is the initial concentration in the basolateral side (μg/mL).

### 2.10. In Vivo Pharmacokinetics of Meloxicam Nanocrystals in Rats

The MLX nanocrystals were suspended in distilled water and orally administrated to rats at a dose of 0.5 mg/kg. Approximately 0.5 mL of the blood sample was collected from the orbit vein at 0, 0.08, 0.25, 0.5, 0.75, 1, 1.5, 2, 4, 6, 12, 24, 48, 72, 96, 120, 144, and 168 h after the MLX nanocrystal administration. All blood samples were centrifuged at 5000 rpm for 5 min at 4 °C, and the plasma was kept at −20 °C until analysis. 

In the sample preparation, a 100 ng/mL piroxicam acetonitrile solution was used as the internal standard (IS), and 50 μL plasma and 10 μL IS solution were placed in a 1.5 mL tube under vortex mixing for 30s; then, 0.5 mL extracting solution (ethyl acetate: isopropanol = 9:1, *v*/*v*) was added under vortex mixing for 3 min. The supernatant organic layer was extracted after centrifuging at 4000 rpm for 5 min, and evaporated by blowing nitrogen at 40 °C to dry. The residue was dissolved by a 400 μL mobile phase, and 100 μL was injected into the LC/MS system. 

For the LC/MS analysis, the chromatographic system consisted of an Agilent 1200–6400; chromatography was conducted using an Agilent column (particle size: 3.5 μm, column dimensions: 2.1 mm × 100 mm), with the column temperature set to 30 °C. The samples were separated using a mobile phase consisting of 10 mM ammonium acetate and acetonitrile (15/85, *v*/*v*) at a flow rate of 0.2 mL/min. Mass spectrometer conditions were dwell time = 200 ms, collision energy = 30 eV, fragmentor energy = 80 eV, gas temperature = 300 °C, gas flow = 6 L/min, and nebulizer = 40 °C. Multiple-reaction-monitoring (MRM) mode was used for quantification by monitoring the transitions. Retention times were 1.02 min for meloxicam (m/z 352→115) and 1.03 min for the internal standard piroxicam (m/z 332→121). Data acquisition and processing were carried out using MassHunter Workstation Software (version B.04.00. Agilent Technologies, Inc., Boulder, CO, USA). The method had a detection limit of 2 ng/mL. The calibration curve was demonstrated to be linear over the concentration range of 2–4000 ng/mL. Both cell and blood samples were analyzed by LC/MS.

### 2.11. Statistical Analysis

Data from the above methods were expressed as mean ± SD, and groups were compared by using Students *t*-tests. Differences were considered statistically significant when *p* < 0.05. Gastroplus^TM^ version 9.8 (Simulations Plus, Inc., Lancaster, CA, USA) was used to analyze the pharmacokinetic data.

## 3. Results and Discussion

### 3.1. Particle Diameter and Morphology Analysis

**Figure 2:**
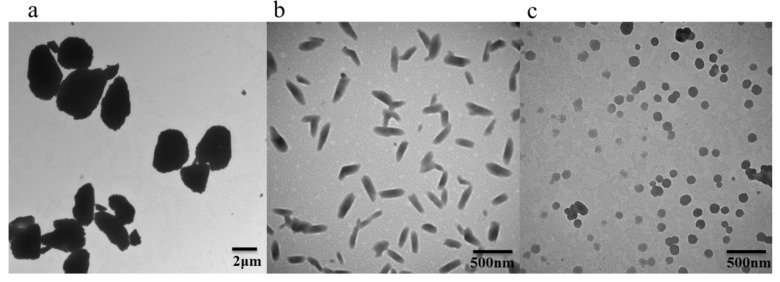
The transmission electron micrographs of nanocrystals: (**a**) MLX-NCs-A; (**b**) MLX-NCs-B; (**c**) MLX-NCs-C.

MLX nanocrystalline suspension particles of different diameters were obtained through wet milling and high-pressure homogenization. As shown in Figure 2, the shape of MLX-NCs-A appeared as an irregular rectangle (Figure 2a). The MLX-NCs-B were observed as long and rodlike (Figure 2b). For the MLX-NCs-C, the shape appeared to be approximately spherical (Figure 2c). 

**Figure 3:**
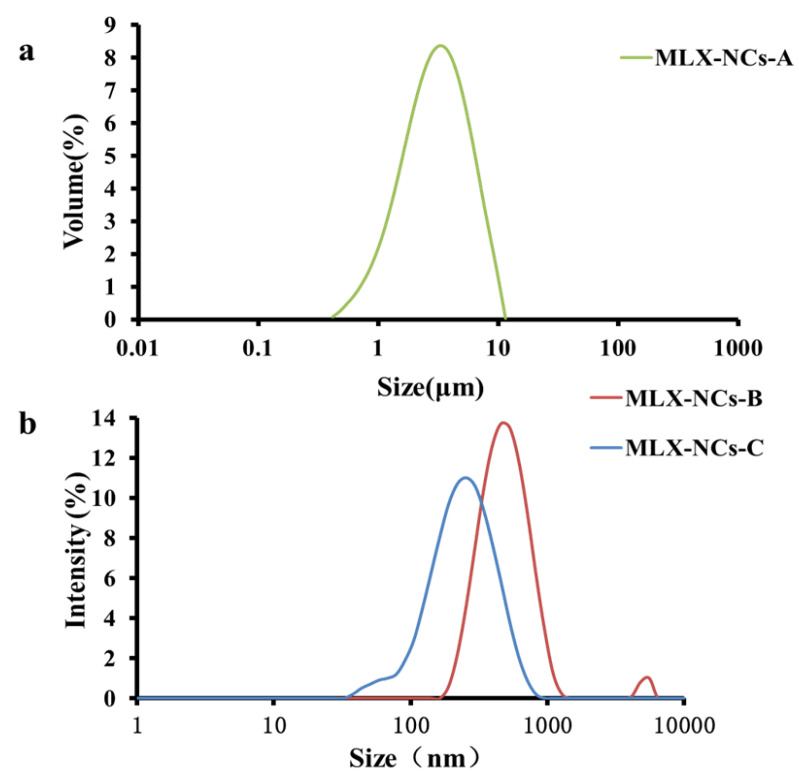
Particle size distributions of (**a**) MLX-NCs-A; (**b**) MLX-NCs-B and MLX-NCs-C.

The particle size measurement results are shown in Figure 3. The sizes of MLX-NCs-A were measured with a Mastersizer 2000 with LS, and the MLX-NCs-B and MLX-NCs-C were measured with a Malvern Zetasizer Nano-ZS90 with DLS. The mean sizes of MLX-NCs-A, MLX-NCs-B and MLX-NCs-C were 3.262 ± 0.016 μm, 460.2 ± 9.5 nm, and 204.9 ± 2.8 nm, respectively. The span of MLX-NCs-A was 1.727 ± 0.011. The PDI values were 0.321 ± 0.021 for MLX-NCs-B and 0.232 ± 0.008 for MLX-NCs-C.

### 3.2. Molecular Simulation

Molecular simulation is a powerful tool used to research the molecular interactions between a stabilizer and drug substance molecules [28]. This technology can explore the interactions between molecules from a microscopic perspective. It can simulate both the static structures of molecules and the dynamic behavior of molecular systems [29]. In the simulation, we regarded MLX and PVP-k17 as a system, and characterized the molecular distributions of MLX and PVP-k17 in water. The hydrogen bonds and the number of molecular contacts among MLX and PVP-k17 were predicted. The van der Waals force and electrostatic interactions were calculated. The mean square displacement (MSD) and root mean square deviation (RMSD) were also calculated. 

**Figure 4:**
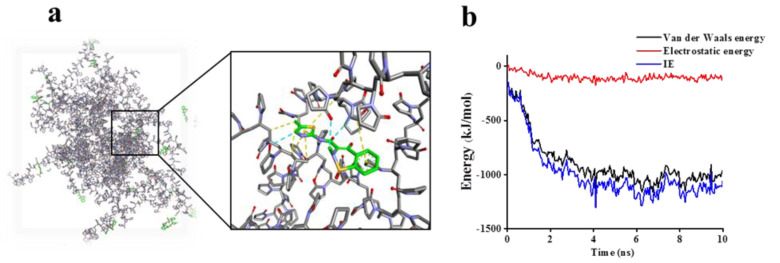
(**a**) The distribution of MLX (green sticks) and PVP-k17 (gray sticks) in the water, hydrophobic interaction (yellow sticks), and hydrogen bonds (light blue sticks); (**b**) the van der Waals energy, electrostatic energy, and interaction energy between MLX and PVP-k17.

According to Figure 4, PVP-k17 was evenly distributed around MLX (Figure 4a), and there were hydrophobic interactions and hydrogen bonds between PVP-k17 molecules and MLX molecules. The interaction energy (IE) between MLX and PVP-k17 included electrostatic interaction and van der Waals energy (Figure 4b). The van der Waals energy was clearly stronger than the electrostatic interaction. IE was governed by van der Waals energy, and reached a stable state after about 4 ns. The van der Waals energy decreased in the first 4 ns, and was at a stable state after 4 ns, with a magnitude of about −1000 kJ/mol. In contrast, the electrostatic interactions were incredibly steady throughout the simulation process, and the magnitude was approximately −100 kJ/mol. The energy between systems tended to be stable after 4 ns. From an energy point of view, the MLX molecules and PVP-k17 molecules were both in a stable system. 

**Figure 5:**
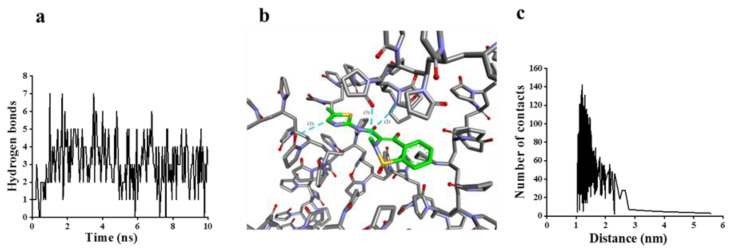
Hydrogen bonds and contact conditions between MLX and PVP-k17. (**a**) Distribution diagram of the number of hydrogen bonds; (**b**) the hydrogen bond formed in the system; (**c**) average number of contacts between MLX and PVP-k17 in the range of 0–6 nm.

The conditions for forming a hydrogen bond are a hydrogen bond distance of ≤ 3.5 nm and a bond angle of close to 180°. MLX constituted of a C=O alkyl hydrogen bond with PVP-k17. The number of hydrogen bonds fluctuated between two and five (Figure 5a). There were three kinds of hydrogen bond in the system: (1) C-H_MLX_…O=C_PVP-k17_; (2) C=O_MLX_…H-C_PVP-k17_; and (3) C-H_MLX_…O=C_PVP-k17_ (Figure 5b). According to Figure 5c, the number of contacts between MLX and PVP-k17 is mainly distributed between 1.0 and 1.5 nm; the number of contacts gradually decreased beyond this distance. When the distance was greater than 3 nm, the number of contacts gradually decreased to close to 0. Therefore, effective hydrogen bond formation and energy reduction were both present in the system, so that PVP-k17 could be successfully wrapped around MLX from the perspectives of time and space. Thus, it can be proven that MLX and PVP-k17 have formed an effective bond [29].

**Figure 6:**
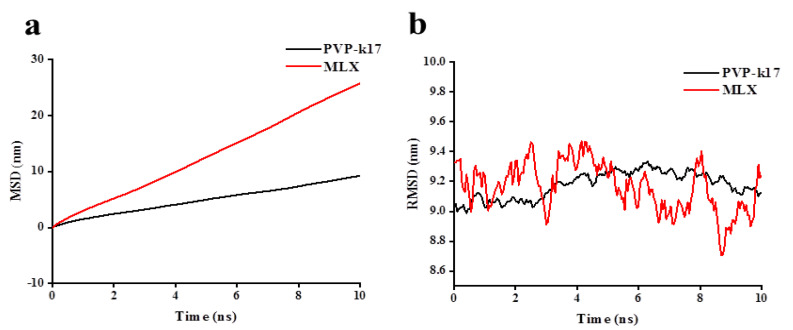
The mean square displacement (**a**) and root mean square deviation (**b**) between MLX and PVP-k17 within 10 ns.

In addition to the analysis above, an investigation of the mobility between molecules was also provided during the simulation. PVP-k17 was relatively stable beyond 10 nm during the simulation, and the MSD changed marginally. However, the MSD of MLX fluctuated greatly below 30 nm (Figure 6a); the corresponding RMSD fluctuated in the range of 8.8–9.4 nm, and PVP-k17 fluctuated in the range of 9.0–9.2 nm (Figure 6b). As the MSD and RMSD results show, MLX was in an unstable state in the water, and the degree of movement within 0–10 ns was relatively large compared to the degree of PVP-k17. The fluctuation trend of MLX was larger than PVP-k17, since the molecular weight of PVP-k17 is almost 20 times that of MLX, and MLX is a small-molecule drug compared with the macromolecular drugs; therefore, its movement in the system is more flexible [30]. Therefore, the fluctuation was within the normal range, and the system tended to be integrally dynamically stable. 

According to the molecule simulation results above, PVP-k17 molecules surrounded the MLX molecules. From the space perspective, the hydrophobic interactions and hydrogen bonds formed between them effectively; from an energy perspective, electrostatic energy and van der Waals energy were generated in the system and changed slightly after 4 ns, thus the system remained in a stable state. Meanwhile, the number of contacts proved that MLX and PVP-k17 molecules formed effective hydrogen bond connections within 10 ns after they contacted, and the number of hydrogen bonds fluctuated between 0 and 7. At the same time, as Figure 5c shows in the number of contacts, effective connections were formed between MLX and PVP-k17 in the range of 1–3 nm, indicating that MLX and PVP-k17 were formed effectively. The MSD value for PVP-k17 was smaller than that for MLX, meaning that PVP-k17 had a lower mobility compared with MLX. Moreover, the RMSD of PVP-k17 had a smaller fluctuation compared with MLX, which further proved that the stability of PVP-k17 in the system was better than MLX. 

### 3.3. Crystalline State Analysis

#### 3.3.1. DSC

**Figure 7:**
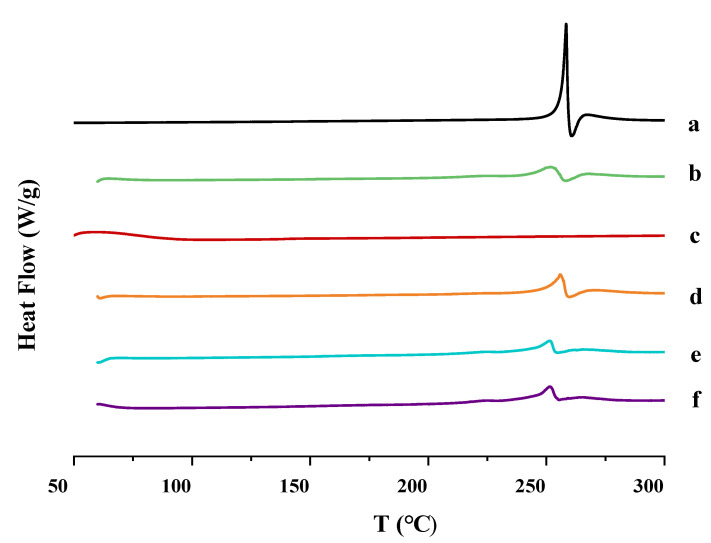
Differential scanning calorimetry graphs (DSC). (**a**) Pure MLX material; (**b**) physical mixture; (**c**) PVP-k17; (**d**) MLX-NCs-A; (**e**) MLX-NCs-B; and (**f**) MLX-NCs-C.

DSC thermograms of the components and the nanocrystal products are shown in Figure 6. The enthalpies of transitions in DSC were showed in Table 1. The pure MLX material exhibited a single sharp melting endothermic peak at 258.38 °C (Figure 7a), corresponding to the melting temperature of MLX [31]. When MLX and PVP-k17 were mixed together, the shape of the melting endothermic peaks compared with MLX was slightly wider and decreased; the endothermic temperature peak of the physical mixture was 251.83 °C (Figure 7b), indicating a certain interaction. The PVP-k17 thermogram revealed no endothermic peak, indicating an amorphous state (Figure 7c). Compared with the mixture, there was little difference between the thermograms of three different particle sizes of nanocrystal: MLX-NCs-A, MLX-NCs-B, and MLX-NCs-C had endothermic peaks at 255.80 °C (Figure 7d), 251.60 °C (Figure 7e), and 251.56 °C (Figure 7f), respectively. The endothermic melting peaks of the nanocrystals were similar with the physical mixture in terms of temperature and shape, and the results showed that the crystalline state and structure of the physical mixture, MLX-NCs-A, MLX-NCs-B, and MLX-NCs-C were similar to each other, although the characteristic peaks were slightly broader compared with pure MLX material, indicating the probable decrease in drug crystallinity. The broadening of the endothermic curves and the decreases in the endothermic peaks were generally caused by the decreased crystallinity in the preparation process [32]. However, the crystallinity of MLX remained in the form of nanocrystals. The PXRD results confirm more information of crystalline state.

#### 3.3.2. PXRD

**Figure 8:**
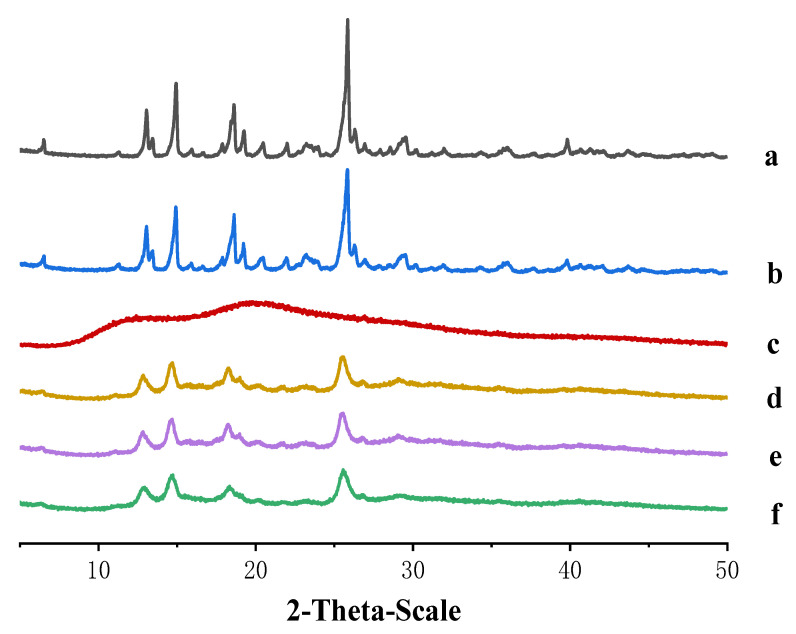
Powder X-ray diffractometry graphs (PXRD). (**a**) Pure MLX material; (**b**) physical mixture; (**c**) PVP-k17; (**d**) MLX-NCs-A; (**e**) MLX-NCs-B; (**f**) MLX-NCs-C.

Powder X-ray diffractometry (PXRD) is an effective technology for the detection of crystalline or amorphous states. The main factors of RXPD are the density, physical hardness, and chemical composition of drugs [33]. The PXRD diagrams of pure MLX material, physical mixture, MLX-NCs-A, MLX-NCs-B, and MLX-NCs-C are shown in Figure 8. The pure MLX material displayed intensive crystalline peaks in the range of 10–50°, with characteristic crystalline peaks at 13.06°, 14.93°, 18.60° and 25.84° at 2θ valve (Figure 8a); MLX can exist five different polymorphic forms. After comparing the PXRD patterns of the five different crystal forms, it was confirmed that the pure MLX material we used is crystal form I [31,34]. When MLX and PVP-k17 were mixed together, the characteristic peaks of mixture were at 13.06°, 14.90°, 18.60° and 25.84° (Figure 8b). There was no obvious difference between the pure MLX materials and the mixture; the diffractogram of the physical mixture is a superposition of its own components. The diffractogram of PVP-k17 had no sharp high-intensity peaks, which showed that it belonged to the amorphous state (Figure 8c). The characteristic peaks of MLX-NCs-A (Figure 8d), MLX-NCs-B (Figure 8e), and MLX-NCs-C (Figure 8f) could be observed in the diffraction pattern, and the sharp peaks were similar to that of pure MLX materials and the mixture. It is easy to cause polymorphic phase transition during the preparation of nanocrystals. In this research, we used high-pressure homogenization to obtain MLX-NCs-A; meanwhile, MLX-NCs-B and MLX-NCs-C were prepared using wet milling technology. Due to the different preparation methods, it was possible to produce polymorphic phase transition. According to the PXRD patterns of three nanocrystals with different particle sizes, their characteristic peaks were extremely similar, and their crystal forms were almost the same. Therefore, the crystallinities of the MLX-NCs-A, MLX-NCs-B, and MLX-NCs-C in our experiment were the same. However, the characteristic peak intensities of three different nanocrystal particle sizes were reduced compared with the pure MLX materials and the mixture, which indicated a decrease in crystallinity during the wet-milling and high-pressure homogenization processes [35]. Combining the results of DSC and PXRD, although crystallinity was decreased in the preparation process, the crystalline state of nanocrystals could still be confirmed. 

### 3.4. Cytotoxicity of the Nanocrystals to Caco-2 Cells

**Figure 9:**
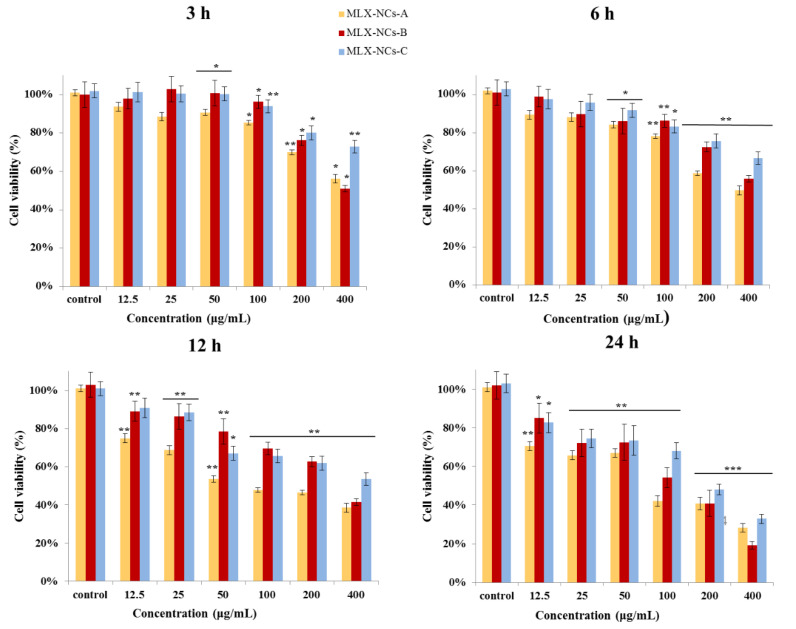
Cell cytotoxicity following MLX-NCs-A, MLX-NCs-B, and MLX-NCs-C treatments for the Caco-2 cell (mean ± SD., n = 6,* *p* < 0.05, ** *p* < 0.01, *** *p* < 0.001 in comparison to the untreated control).

The cytotoxicity of MLX nanocrystals of different particle sizes was evaluated in the Caco-2 cell line at 3, 6, 12, and 24 h. Figure 9 shows the cell viability of Caco-2 in different concentrations of MLX-NCs-A, MLX-NCs-B, and MLX-NCs-C. Cytotoxicity is influenced by the drug concentrations and culture times. The higher the drug concentration, the longer the culture time and the greater the number of inhibited cells [36]. We found that under the same concentrations and culture times, reducing the particle size of nanocrystals would decrease the cytotoxicity. The results showed that cell viability was not only decreased in time- and dose-dependent manners when Caco-2 cells were exposed to three different particle sizes of MLX nanocrystals, but also correlated with the particle size [37]. The cytotoxicity increased with the increasing particle size. The cell viabilities of MLX-NCs-A, MLX-NCs-B, and MLX-NCs-C were 70%, 76%, and 80% at a concentration of 200 μg/mL in 3 h. After reducing the administration concentration to 100 μg/mL, the cell viability of MLX-NCs-A, MLX-NCs-B, and MLX-NCs-C were all above 85%. Additionally, based on this result, we set the administration concentration of transwells to below 100 μg/mL. Meanwhile, when the concentration was increased to 400 μg/mL, the cytotoxicity was significantly increased, and the cell viabilities were 56%, 51%, and 73% for MLX-NCs-A, MLX-NCs-B, and MLX-NCs-C, respectively. At the same concentration, as the incubation time increased, the cytotoxicity also increased significantly. The cell viabilities of MLX-NCs-A, MLX-NCs-B, and MLX-NCs-C were decreased to 50%, 56%, and 67% in 6 h, respectively. When the incubation time was for 12 h, the cell viabilities of MLX-NCs-A, MLX-NCs-B, and MLX-NCs-C were 38%, 41%, and 53%, respectively; however, when the culture time was for 24 h, the cell viabilities were all reduced to below 40%. In this process, we found that, as the size of the nanocrystals decreased, the toxicity to Caco-2 cells was also reduced. In the three different nanocrystal particle sizes, MLX-NCs-C were the smallest, and its cytotoxicity was also the lowest under the same culture condition. A smaller particle size is beneficial for the dissolution of drug, which can improve the absorption and additionally directly aid the cellular uptake of nanocrystals [38]; we speculated that this was the reason why a decreased particle size could reduce cytotoxicity. The particle size of MLX-NCs-A was 3.262 ± 0.016 μm, and it dissolved slowly, which affected the absorption of the drug, and the larger particle size affected the direct uptake of nanocrystals by the cells [39]. 

### 3.5. In Vitro Transport Studies of the Caco-2 Cell Model

**Figure 10:**
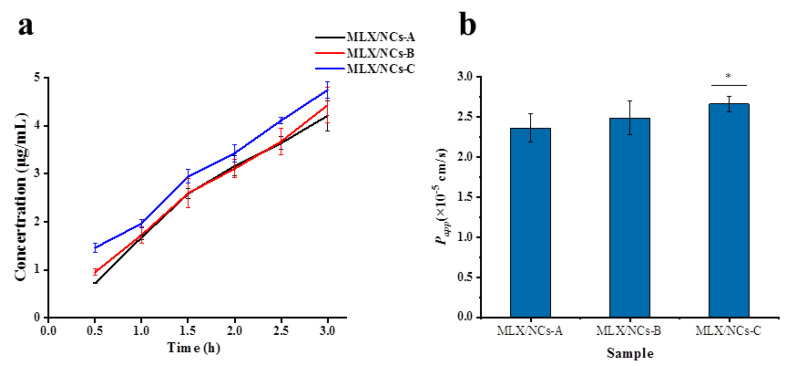
Cumulative amounts and *P_app_* of MLX-NCs-A, MLX-NCs-B, and MLX-NCs-C transport across Caco-2 cell monolayers (mean ± SD., *n* = 4). (**a**) Curve of transport amount over time within 3 h (**b**) the permeability coefficient (*P_app_*) at 3 h for MLX-NCs-A, MLX-NCs-B, and MLX-NCs-C, * *p* < 0.05 in comparison to the MLX-NCs-A.

The Caco-2 cell model has been widely used in transport studies due to its similar characteristics to the intestinal epithelium [40]. A TEER value between 200 and 1000 Ω/cm^2^ and *P_app_* ≤ 2 × 10^−6^ cm/s represents an integral cell transport model [41,42]. Figure 10 shows the time course of the MLX-NCs-A, MLX-NCs-B, and MLX-NCs-C cumulative transport across Caco-2 monolayers. The transport fluxes were 4.21, 4.43, and 4.74 μg/mL at 3 h for MLX-NCs-A, MLX-NCs-B, and MLX-NCs-C, respectively. The *P_app_* values of MLX-NCs-A, MLX-NCs-B, and MLX-NCs-C were 2.37, 2.49, and 2.67 × 10^−^^5^ cm/s, respectively. MLX belongs to the BCS II category; its high permeability was demonstrated here. The transport amounts of nanocrystals with three different particle sizes exhibited differences in the initial 15 min and after 3 h; however, from 1.0 h to 2.5 h, the transport amounts of MLX-NCs-A and MLX-NCs-B were almost same. The smaller particle size presented increased transport capacity under the same conditions [43], but the differences between MLX-NCs-A and MLX-NCs-B were not obvious. However, the results of the *P_app_* increased with size-dependent decreases in the MLX particles, which was consistent with our expectations.

### 3.6. In Vivo Transdermal Delivery

**Figure 11:**
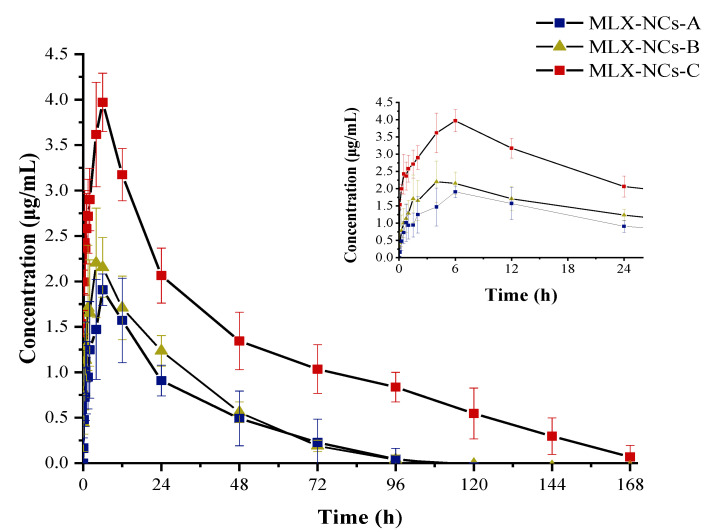
Plasma concentration–time curves of MLX-NCs-A, MLX-NCs-B, and MLX-NCs-C in rats after oral administration (mean ± SD., MLX-NCs-A and MLX-NCs-B n = 7; MLX-NCs-C n = 6).

Nanocrystals can increase the appearance solubility and dissolution of MLX [20]; moreover, the safety and effectiveness of MLX nanocrystals used in oral preparations is vital. Therefore, we used three different particle sizes of nanocrystals for in vivo experiments, and calculated the relevant pharmacokinetic parameters. Figure 11 shows the plasma concentration–time curve of MLX-NCs-A, MLX-NCs-B and MLX-NCs-C in rats after oral administration (0.5 mg/kg). Relevant pharmacokinetic parameters, including AUC_0–∞,_ t_1/2_, *K*_a_, MRT, AUMC, *C*_max_ and *T*_max_, are listed in Table 2.

Compared with the pharmacokinetic parameters of three different particle sizes of MLX nanocrystals, when MLX was completely eliminated, the AUC_0–∞_ of MLX/NCs-A, MLX/NCs-B, and MLX/NCs-C were 55.20, 67.72, and 197.58 μg h/mL, respectively, and the AUC_0–∞_ value of MLX/NCs-C was 3.58- and 2.92-fold higher than those of MLX/NCs-A and MLX/NCs-B. The C*_max_* value of MLX/NCs-C was 2.07- and 1.77-fold greater than those of MLX/NCs-B and MLX/NCs-C, respectively. For the t_1/2_, MLX/NCs-C increased by 1.0 h and 0.34 h over MLX/NCs-A and MLX/NCs-B, respectively. Through comparison of the pharmacokinetic parameters of three nanocrystals of different particle sizes, it was demonstrated that they exhibited a better trend as the particle size decreased, the t_1/2_ was extended, and AUC_0–∞_ also increased significantly. The adhesion of drug nanocrystals in the gastrointestinal tract was increased, which could also better increase the residence time and increase the bioavailability of the drug [44]. According to the particle size, the size of MLX-NCs-A was micron-level, the size of MLX-NCs-C was nanometer level, and the size of MLX-NCs-B was generally to be sub-micron. For BCS II drugs, dissolution is an important factor limiting absorption in vivo. Significant reductions in particle sizes can increase the dissolution rate and improve the bioavailability of the drug [37]. A smaller particle size can increase AUC_0–∞_; the t_1/2_ and MRT values will increase accordingly. This is also consistent with previous reports [23,25,45]. However, the differences in pharmacokinetic parameters of MLX-NCs-A and MLX-NCs-B were not obvious, and the differences between MLX-NCs-B and MLX-NCs-C were significant, which was identified in our transport study results. We hypothesize that there is a critical particle size (CPS), measured between the nanometer and sub-micron levels, which can determine the change in related pharmacokinetic behavior. When the particle size of the nanocrystal is larger than the CPS, it will not affect the pharmacokinetic behavior; when the particle size is smaller than the CPS, the pharmacokinetic parameters will change.

## 4. Conclusions

Nanocrystal technology is an effective method to improve the apparent solubility, dissolution, and oral bioavailability for poorly water-soluble drugs. In this study, we prepared three different particle sizes of MLX nanocrystals through wet-milling and high-pressure homogenization to explore the influence of particle size in vivo and in vitro. Analysis of the crystalline state showed no effect on the crystallization of nanocrystals during the preparation process. Molecular simulations demonstrated the interactions and energy generated between MLX and PVP-k17, indicating that they formed effective connections. The results of the cytotoxicity and transport studies also proved that as the particle size decreased, the cell viability and transportation efficiency increased. In addition, the in vivo pharmacokinetic studies of MLX-NC in rats showed that the oral bioavailability increased as the particle size decreased. Therefore, nanocrystal technology is a feasible method to improve poorly water-soluble drugs, and can solve many related problems in order to develop oral-insoluble drug formulations.

## Figures and Tables

**Table 1:** The enthalpy values in the DSC transitions.

	Pure MLX Material	Physical Mixture	PVP-k17	MLX-NCs-A	MLX-NCs-B	MLX-NCs-C
Enthalpy (J/g)	129.0	71.5	41.8	13.8	12.9	12.3

**Table 2:** Pharmacokinetic parameters after the oral administration of MLX-NCs-A, MLX-NCs-B, and MLX-NCs-C in rats. (0.5 mg/kg, mean ± SD, MLX-NCs-A and MLX-NCs-B n = 7; MLX-NCs-C n = 6).

	MLX-NCs-A	MLX-NCs-B	MLX-NCs-C
AUC_0–∞_ (μg h/mL)	55.20 ± 16.53 ^ΔΔΔ^	67.72 ± 9.34 ***	197.58 ± 30.90
*t*_1/2_ (1/h)	23.32 ± 10.45 ^Δ^	20.00 ± 2.10 ***	44.78 ± 8.18
*C_max_ (*μg/mL)	1.90 ± 0.17 ^ΔΔΔ^	2.20 ± 0.60 ***	3.97 ± 0.32
*T*_max_ (*h*)	4.67 ± 1.50	5.33 ± 0.94 ***	5.67 ± 0.75
*K*_a_ (1/h)	1.22 ± 1.03 ^ΔΔ^	2.35 ± 1.93 ***	6.80 ± 1.07
MRT (h)	33.64 ± 15.07 ^ΔΔΔ^	28.85 ± 3.03 ***	60.98 ± 11.64
AUMC (h^2^ μg/mL)	2591.33 ± 1607.43 ^ΔΔΔ^	1998.97 ± 318.74 ***	14234.53 ± 4810.52

^Δ^ *p* < 0.05 ^ΔΔ^ *p* < 0.01 ^ΔΔΔ^ *p* < 0.001 vs. the parameters of MLX-NCs-C. *** *p* < 0.001 vs. the parameters of MLX-NCs-C.

## Data Availability

All data are included at the manuscript.

## Sample Availability

Samples of the compounds are available from the authors.

## References

[1] Cheney M.L., Weyna D.R., Shan N., Hanna M., Wojtas L., Zaworotko M.J. (2011). Coformer selection in pharmaceutical cocrystal development: A case study of a meloxicam aspirin cocrystal that exhibits enhanced solubility and pharmacokinetics. J. Pharm. Sci..

[2] Meng L., Mohammad A., Rajesh D., Ecevit B.J.P. (2016). Nanomilling of Drugs for Bioavailability Enhancement: A Holistic Formulation-Process Perspective. Pharmaceutics.

[3] Luger P., Daneck K., Engel W., Trummlitz G., Wagner K.J.E. (1996). Structure and physicochemical properties of meloxicam, a new NSAID. Eur. J. Pharm. Sci..

[4] Ambrus R., Kocbek P., Kristl J., Šibanc R., Rajkó R., Szabó-Révész P. (2009). Investigation of preparation parameters to improve the dissolution of poorly water-soluble meloxicam. Int. J. Pharm..

[5] Leuner C., Dressman J.J.E. (2000). Improving drug solubility for oral delivery using solid dispersions. Eur. J. Pharm. Biopharm..

[6] Suzuki H., Yakushiji K., Matsunaga S., Yamauchi Y., Seto Y., Sato H., Onoue S.J. (2017). Amorphous solid dispersion of meloxicam enhanced oral absorption in rats with impaired gastric motility. J. Pharm. Sci..

[7] Peltonen L., Hirvonen J.J. (2010). Pharmaceutical nanocrystals by nanomilling: Critical process parameters, particle fracturing and stabilization methods. J. Pharm. Pharmacol..

[8] Elita S., Janaine C., Fernanda T., Juliana A., Gabriela D., Fátima B., Francisco P., Adriana L., Ana H., Drugs C.L.J.I.N. (2013). Ketoprofen-loaded polymeric nanocapsules selectively inhibit cancer cell growth in vitro and in preclinical model of glioblastoma multiforme. Investig. New Drugs.

[9] Raffin R.P., Lima A., Lorenzoni R., Antonow M.B., Turra C., Alves M.P., Fagan S.B.J. (2012). Natural lipid nanoparticles containing nimesulide: Synthesis, characterization and in vivo antiedematogenic and antinociceptive activities. J. Biomed. Nanotechnol..

[10] Ianiski F.R., Alves C.B., Souza A., Pinton S., Luchese C.J. (2012). Protective effect of meloxicam-loaded nanocapsules against amyloid-β peptide-induced damage in mice. Behav. Brain Res..

[11] Bernardi A., Frozza R.L., Horn A.P., Campos M.M., Battastini A.J.N.I. (2010). Protective effects of indomethacin-loaded nanocapsules against oxygen-glucose deprivation in organotypic hippocampal slice cultures: Involvement of neuroinflammation. Neurochem. Int..

[12] Bernardi A., Zilberstein A., Jger E., Campos M.M., Morrone F.B., Calixto J.B., Pohlmann A.R., Guterres S.S., Battastini A.J.B. (2009). Effects of indomethacin-loaded nanocapsules in experimental models of inflammation in rats. Br. J. Pharmacol..

[13] Lu Y., Qi J., Dong X., Zhao W., Wu W.J. (2017). The in vivo fate of nanocrystals. Drug Discov. Today.

[14] Pawar V.K., Singh Y., Meher J.G., Gupta S., Chourasia M.K.J. (2014). Engineered nanocrystal technology: In-vivo fate, targeting and applications in drug delivery. J. Control. Release.

[15] Gujar K., Wairkar S.J.P. (2020). Nanocrystal technology for improving therapeutic efficacy of flavonoids. Phytomedicine.

[16] Van Eerdenbrugh B., Vermant J., Martens J.A., Froyen L., Van Humbeeck J., Augustijns P., Mooter G.V.D. (2009). A screening study of surface stabilization during the production of drug nanocrystals. J. Pharm. Sci..

[17] Keck C.M., Muller R.H. (2006). Drug nanocrystals of poorly soluble drugs produced by high pressure homogenisation. Eur. J. Pharm. Biopharm..

[18] Langguth P., Hanafy A., Frenzel D., Grenier P., Nhamias A., Ohlig T., Vergnault G., Spahn-Langguth H.J. (2005). Nanosuspension Formulations for Low-Soluble Drugs: Pharmacokinetic Evaluation Using Spironolactone as Model Compound. Drug Dev. Ind. Pharm..

[19] Malamatari M., Taylor K.M., Malamataris S., Douroumis D., Kachrimanis K. (2018). Pharmaceutical nanocrystals: Production by wet milling and applications. Drug Discov. Today.

[20] Ochi M., Kawachi T., Toita E., Hashimoto I., Yuminoki K., Onoue S., Hashimoto N.J. (2014). Development of nanocrystal formulation of meloxicam with improved dissolution and pharmacokinetic behaviors. Int. J. Pharm..

[21] Bolourchian N., Nili M., Foroutan S.M., Mahboubi A., Nokhodchi A.J. (2019). The use of cooling and anti-solvent precipitation technique to tailor dissolution and physicochemical properties of meloxicam for better performance. J. Drug Deliv. Sci. Technol..

[22] Mauludin R., Müller R.H., Keck C.M.J.E. (2009). Kinetic solubility and dissolution velocity of rutin nanocrystals. Eur. J. Pharm. Sci..

[23] Xia D., Cui F., Piao H., Cun D., Piao H., Jiang Y., Mei O., Quan P.J. (2010). Effect of Crystal Size on the In Vitro Dissolution and Oral Absorption of Nitrendipine in Rats. Pharm. Res..

[24] Tu L., Yi Y., Wu W., Hu F., Hu K., Feng J.J. (2013). Effects of particle size on the pharmacokinetics of puerarin nanocrystals and microcrystals after oral administration to rat. Int. J. Pharm..

[25] Sun J., Wang F., Sui Y., She Z., Zhai W., Wang C., Deng Y. (2012). Effect of particle size on solubility, dissolution rate, and oral bioavailability: Evaluation using coenzyme Q10 as naked nanocrystals. Int. J. Nanomed..

[26] Jinno J.I., Kamada N., Miyake M., Yamada K., Mukai T., Odomi M., Toguchi H., Liversidge G.G., Higaki K., Kimura T. (2006). Effect of particle size reduction on dissolution and oral absorption of a poorly water-soluble drug, cilostazol, in beagle dogs. J. Control. Release.

[27] Kilkenny C., William B.J., Cuthill I.C., Emerson M., Altman D.G. (2010). Improving bioscience research reporting: The ARRIVE guidelines for reporting animal research. PLoS Biol..

[28] Cui Y.J. (2011). Using molecular simulations to probe pharmaceutical materials. J. Pharm. Sci..

[29] Yani Y., Chow P.S., Tan R.B.H.J. (2011). Molecular simulation study of the effect of various additives on salbutamol sulfate crystal habit. Mol. Pharm..

[30] Price D.J., Iii C.J. (2002). Modern protein force fields behave comparably in molecular dynamics simulations. J. Comput. Chem..

[31] Freitas J., Viana O., Bonfilio R., Doriguetto A.C., Sciences M.A. (2017). Analysis of polymorphic contamination in meloxicam raw materials and its effects on the physicochemical quality of drug product. Eur. J. Pharm. Sci..

[32] Freag M.S., Elnaggar Y., Abdallah O.Y.J. (2013). Development of novel polymer-stabilized diosmin nanosuspensions: In vitro appraisal and ex vivo permeation. Int. J. Pharm..

[33] Müller R., Jacobs C., Kayser O.J. (2001). Nanosuspensions as particulate drug formulations in therapy. Rationale for development and what we can expect for the future. Adv. Drug Deliv. Rev..

[34] Coppi L., Sanmarti M.B., Clavo M.C. (2005). Crystalline Forms of Meloxicam and Processes for Their Preparation and Interconversion. U.S. Patent.

[35] Monteiro A., Afolabi A., Bilgili E. (2012). Continuous production of drug nanoparticle suspensions via wet stirred media milling: A fresh look at the Rehbinder effect. Drug Dev. Ind. Pharm..

[36] Di J., Gao X., Du Y., Zhang H., Zheng A.J.A. (2020). Size, shape, charge and stealthy surface: Carrier properties affect the drug circulation time in vivo. Asian J. Pharm. Sci..

[37] Bi C., Miao X.Q., Chow S.F., Wu W.J., Yan R., Liao Y.H., Chow A.H., Zheng Y. (2017). Particle size effect of curcumin nanosuspensions on cytotoxicity, cellular internalization, in vivo pharmacokinetics and biodistribution. Nanomed. Nanotechnol. Biol. Med..

[38] Dausend J., Musyanovych A., Dass M., Walther P., Schrezenmeier H., Landfester K., Bioscience V.M.J.M. (2008). Uptake Mechanism of Oppositely Charged Fluorescent Nanoparticles in HeLa Cells. Macromol. Biosci..

[39] Adjei I.M., Sharma B., Labhasetwar V.J.S.N. (2014). Nanoparticles: Cellular uptake and cytotoxicity. Adv. Exp. Med. Biol..

[40] Bailey C.A., Piotr B., Malick Waseem A. (1996). The use of the intestinal epithelial cell culture model, Caco-2, in pharmaceutical development. Adv. Drug Deliv. Rev..

[41] Senem K., Esra C., Charlotte G., John V.C.J.I. (2015). Anthocyanin Absorption and Metabolism by Human Intestinal Caco-2 Cells—A Review. Int. J. Molec. Sci..

[42] Sun H., Chow E.C., Liu S., Du Y., Pang K.S. (2008). The Caco-2 cell monolayer: Usefulness and limitations. Expert Opin. Drug Metab. Toxicol..

[43] Win K.Y., Feng S.S.J. (2005). Effects of particle size and surface coating on cellular uptake of polymeric nanoparticles for oral delivery of anticancer drugs. Biomaterials.

[44] Rabinow B.E.J. (2005). Nanosuspensions in drug delivery. Nat. Rev. Drug Discov..

[45] Imono M., Uchiyama H., Yoshida S., Miyazaki S., Tozuka Y.J.E. (2019). The elucidation of key factors for oral absorption enhancement of nanocrystal formulations: In vitro—in vivo correlation of nanocrystals. Eur. J. Pharm. Biopharm..

